# Progress on Suzuki–Miyaura Cross-Coupling Reactions Promoted by Palladium–Lanthanide Coordination Polymers as Catalytic Systems

**DOI:** 10.3390/molecules31020378

**Published:** 2026-01-21

**Authors:** Fu Ding, Ileana Dragutan, Lixin You, Yaguang Sun, Valerian Dragutan

**Affiliations:** 1 The Key Laboratory of Inorganic Molecule-Based Chemistry of Liaoning Province, Shenyang University of Chemical Technology, Shenyang 110142, China; youlx@syuct.edu.cn; 2 “C.D. Nenitzescu” Institute of Organic and Supramolecular Chemistry, Romanian Academy, 202B Spl. Independentei, 060023 Bucharest, Romania; mdragutan@gmail.com

**Keywords:** lanthanide organic frameworks, palladium heterogeneous catalysts, Suzuki–Miyaura cross-couplings, NHC ligands, coordination polymers, MOFs

## Abstract

Lanthanide coordination polymers have been developed at a fast rate during the past two decades due to their appealing applications in the modern field of materials science and emerging technologies like luminescence, magnetism, sensing, gas adsorption, and catalysis. The role of lanthanides in imparting specific properties to the coordination polymers has been fully documented in extensive studies carried out by numerous research groups. It has been shown that because lanthanide(III) ions possess a variable coordination number, they readily build two-dimensional and three-dimensional architectures with definite channels, permanent pores, and distinct surface areas. Due to their strong oxophilic propensity and hard Lewis acid character, lanthanides favor the construction of stable coordination polymers and MOF configurations by strongly binding the coordinating groups of the organic linkers. Associated with palladium complexes, the lanthanide ions provide synergistic effects with Lewis acid sites, beneficial to the catalytic activity. These attractive characteristics of lanthanides enabled them to be fruitfully applied in Pd-Ln coordination polymers with catalytic properties. This review covers an array of Pd-Ln coordination polymers applied as heterogeneous catalysts in Suzuki–Miyaura C(sp^2^)-C(sp^2^) cross-coupling reactions. The activity and chemoselectivity of Pd(II) ions and Pd nanoparticles associated in coordination polymers with different lanthanides from a selected array of rare earth elements (Eu, Sm, Eu, Gd, Pr, Nd, Ce, La, or Tb) is discussed. High yields (>99%) are attained under optimized reaction conditions. The specific role of lanthanides and organic ligands in creating sustainable and recyclable heterogeneous Pd catalysts is evidenced. Mechanistic aspects of the C(sp^2^)-C(sp^2^) cross-coupling reactions are considered. The synergistic interaction between lanthanides and palladium as well as with the organic ligands is highlighted.

## 1. Introduction

Carbon–carbon bond-forming reactions are well-established chemical transformations in synthetic organic chemistry. By this strategy, the production of a wide platform of multifunctional organic compounds has become possible [1,2,3]. Many of these versatile catalytic transformations promoted by palladium catalytic systems such as Suzuki–Miyaura [4,5,6], Mizoroki–Heck [7,8], Sonogashira [9], Kumada–Tamao–Corriu [10], Stille [11,12,13], Negishi [14], and Hiyama [15] cross-coupling reactions have been successfully applied to construct new complex organic molecules. The broad range of organic compounds obtained by these methods includes substituted biphenyls, styrene, stilbenes, olefins, or acetylenes and expands to intricate organic compounds and intermediates useful in the synthesis of a diversity of fine chemicals, agrochemicals, pharmaceuticals, petrochemicals, bioactive natural products, and nanomaterials [16,17]. Since its discovery, the Suzuki–Miyaura [5] C(sp^2^)-C(sp^2^) cross-coupling reaction (Equation (1)) has played a prominent role in the development of this resourceful and productive synthetic strategy by an inherent diversification of the substrates, catalysts, reaction conditions, and organic compounds obtained [16,17,18].


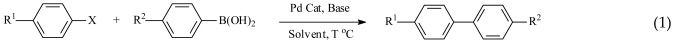


In this respect, the catalytic systems have been extended to new palladium complexes applied in homogeneous and heterogeneous phases and to new transition metal compounds like Ni, Cu, Fe, and Ru, using either conventional or more sophisticated phosphine ligands, diversified organic ligands, ancillary dendritic, and NHC ligands. In addition to their availability, performant catalytic activity and selectivity, and their low-cost as compared to conventional palladium systems, the broad array of newly developed transition metal-based catalysts offer further advantages by their effective application to new substrates like aryl ethers, esters, phosphates, fluorides, phenols, and heteroatom-containing compounds, which are of a practical utility for synthesis of fine chemicals and pharmaceuticals [16]. As compared to previous palladium catalytic systems, a modern trend involves the immobilization of palladium complexes on a wide range of solid supports. This concept has been successfully applied to Pd-Ln coordination polymers, as will be shown below. Considerable progress has been made by optimizing the reaction conditions (solvent, base, catalyst loading, and temperature) and extending the initially used organic solvents to aqueous systems. Moreover, advancements have been made in using very low catalyst loadings (<1 mol%) and no additional ligands, which is essential for eco-friendly applications on a larger scale [19].

At this time, a broad array of Pd complexes, as well as complexes of other transition metals, mostly in the presence of a base, are currently employed as efficient, chemoselective homogeneous or heterogeneous catalysts to promote carbon–carbon cross-coupling reactions [18,19,20,21,22,23,24,25,26,27,28,29]. In the homogeneous phase, the utilization of aqueous media has recently enjoyed sustained development in order to satisfy the multiple environmentally benign requirements of the present technological trends [30,31,32]. In this context, of special merit is the application of Suzuki–Miyaura cross-coupling reaction in neat water, leading to many beneficial results in process application and from an environmental point of view [30]. On the other hand, heterogeneous Pd catalytic systems have seen a major development generated by the upper-level capabilities of the novel supports in finely tuning the catalyst activity, chemoselectivity, and, of great practical significance, the stability, regeneration, and recycling of the catalytic system [33,34,35,36,37,38,39,40,41,42,43,44,45,46,47,48,49,50,51,52,53,54,55,56,57,58,59,60,61,62,63,64,65,66,67,68,69,70,71,72,73]. Novel attractive methodologies have been developed to address the ample scope of this catalytic process including the utilization of palladium nanoparticles [33,34,35,36,37,38], palladium immobilized on magnetic nanoparticles [39], or natural supports [40,41]. Noticeable progress in this field concerns the utilization of nucleophilic carbene ligands (mostly NHCs) [19,42,43,44,45,46,47,48], Schiff bases [49,50], and water-soluble ligands such as poly(ethylene glycol)-functionalized N-heterocyclic carbenes [51]. New ligands like thiourea [52] or phosphines [53,54] have also been employed. Furthermore, progress has been made on activation by microwave (MW) acceleration [55,56,57,58], the utilization of “greener” solvents such as water systems [30,59,60,61,62,63,64,65,66,67,68,69,70,71,72,73] (in the presence of MW), and catalysis under micellar environment for enhancing the solubility of the aromatic halides. Special attention was paid to mixed reaction media such as water–ethanol or water–DMF [71] and to palladium complexes partially associated with ionic liquids [72]. Related to these advancements, carbon–carbon cross-coupling reactions have long been the subject of a vast body of research in organic synthesis and transition-metal catalysis [73]. Their design contributed to highly advanced applications for catalyst efficiency, sustainable “greener” processes, and environmental protection [73]. Within this area, new complexes of various transition metals have been discovered [74,75,76] and employed as homogeneous and heterogeneous catalysts for cross-coupling reactions. Special attention was paid to Ni catalysts discovered by Percec [74,75,76] which, despite needing a larger amount of catalyst loading, are affordable, inexpensive, and could be applied to a broader array of organic substrates [77,78,79,80]. Furthermore, heterogeneous Pd catalysts have been consistently upgraded by implementing a large variety of robust, solid supports that impart to the catalytic systems unprecedented high activity and stability [81,82,83,84,85,86,87,88,89,90,91,92,93,94,95,96,97,98,99,100,101,102,103,104,105,106,107,108,109,110]. These advancements widened the perspectives for productive applications of the new palladium catalytic systems on a large scale.

Following the recent achievements in cross-coupling reactions induced by palladium catalytic systems, in this work we survey the most relevant advancements in cross-couplings performed with Pd-Ln coordination polymers as catalysts, mainly during the past decade.

## 2. Development of Pd-Ln Coordination Polymers: The Role of Lanthanides

Promoting the innovative development of coordination polymers, lanthanides [111,112,113], due to their unique electronic configuration, strong oxophilic propensity, and hard Lewis acid character, effectively succeeded in generating stable, multifunctional coordination polymers (CPs) and metal–organic frameworks (MOFs) as efficient supports for Pd catalysts. The lanthanides are incorporated into one-, two-, and three-dimensional coordination polymers by means of the coordinating groups of the organic linkers [114]. The variable ionic radii allow different coordination numbers, generating new, totally unusual structures and matrices with distinct physical–chemical properties [115]. Moreover, since in lanthanides the 4f orbitals are buried into the core and are well shielded by the outer filled 5s and 5p shells, in a totally different manner from the transition d metal ions, the coordination chemistry of Ln ions is governed by entropy effects and not by orbital directionality like transition metals [89]. This fact has important consequences for the stability of different configurations built with the participation of lanthanides. It has been shown that owing to the variable coordination numbers, lanthanide ions form coordination complexes with a wide range of coordination modes and network geometries [89]. The coordination numbers and the geometries depend greatly on the steric hindrance of the coordinating ligands. This fact will result in a broad diversity of possible coordination modes and network architectures of the Ln coordination polymers. The distinct nature of the lanthanide and the organic linker bridging the lanthanide nodes governs the stability of the coordination polymers, the size and distribution of the matrix pores, the number and dimensions of the polymer channels, the surface properties, and the related structural parameters, which are crucial for their practical applications. In addition to their valuable luminescent [89,90,91,116,117,118,119,120], magnetic [121,122], sensing [91,117], and gas adsorption and separation [101] properties, the new Ln-CPs and Ln-MOFs containing a variety of lanthanides in their matrix unit serve as adequate templates for emerging applications [97]. In this context, due to their multifunctional ability, lanthanides succeed in greatly modulating the catalytic properties of the tethered active Pd complexes by their synergistic interaction with the catalytically active sites through the connecting organic ligands.

**Scheme 1:**
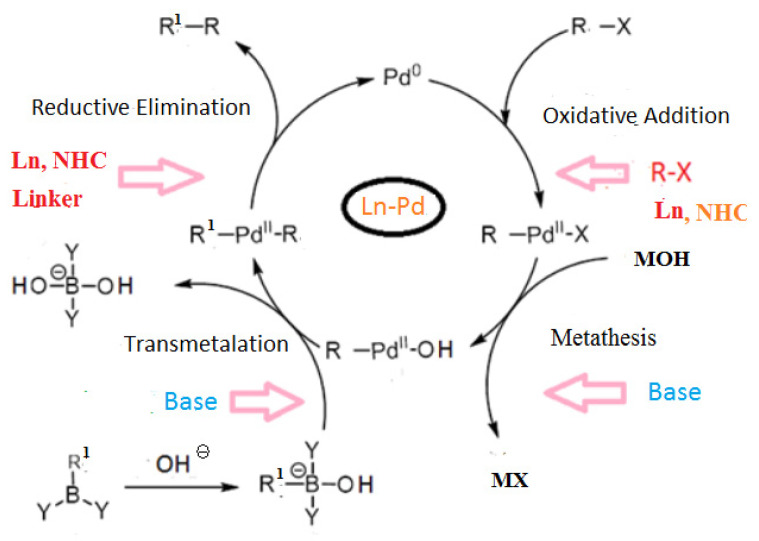
Pd catalytic cycle for Suzuki–Miyaura cross-coupling with Pd-Ln coordination polymers.

It has been firmly evidenced that lanthanides incorporated into coordination polymers cooperate with palladium active sites and will thus increase the activity and chemoselectivity of the catalytic system. Due to the fact that lanthanides display higher coordination abilities, they are able to bridge a large number of metal cations, increasing the metal density with a beneficial catalytic outcome. In this way, they also permit the introduction of multiple functionalities in the heterometallic polymers. As a consequence, this process will allow the generation of a diversity of active sites on the surface of the catalyst. Such composite materials result eventually in high activity and selectivity of the catalytic cross-coupling reactions. It has been fully documented that due to their electronic configuration, lanthanides influence the coordinative palladium bonds, enhancing the back-donation charge contribution, providing in this manner rather stable Pd complexes. Moreover, taking into account their pronounced electropositive propensity, lanthanides will influence considerably the oxidative addition and reductive elimination steps of the palladium catalytic cycle (Scheme 1; Equation (2)). This notable cooperation of lanthanides with palladium catalysts is further enhanced through the steric and electronic effects of the ancillary ligands of palladium such as N-heterocyclic carbenes.


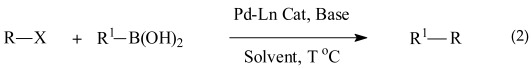


## 3. Design of Organic Ligands/Linkers for Pd-Ln Coordination Polymers

Experimental results indicated that the organic ligands associated with lanthanides and palladium play an important role in creating sustainable Pd-Ln heterogeneous catalytic systems. There are two classes of organic ligands that are currently used in the Pd-Ln coordination polymers applied as catalysts in C(sp^2^)-C(sp^2^) cross-coupling reactions. The first type consists of organic ligands or linkers designed to bridge the lanthanides, building in this way the sterical pattern of one-, two-, and three-dimensional coordination polymers. Moreover, they are suitable to coordinate with the palladium ions, helping to anchor and conveniently distribute the palladium active sites on the surface of the heterogeneous catalyst. Consequently, the organic ligands coordinating the lanthanides and palladium played multiple roles in activating the Pd-Ln containing catalysts for the Suzuki–Miyaura reaction. Owing to the special ability of the carboxylate-type ligands to coordinate Ln ions, they effectively succeed in building polymers with a definite configuration endowed with high stability of the unit pattern, pronounced robustness, large surface area, and uniform pore size distribution, which are necessary for applications on a large scale [123,124,125,126,127,128,129,130,131,132,133,134,135,136,137,138,139,140,141,142,143,144,145,146,147,148,149] (Table 1).

The stability of the polymers is the result of the strong oxophilic character of Ln ions that prefer coordination with nucleophilic carboxyl ligands. Such ligands allow monodentate, bidentate, or multidentate coordination modes of lanthanides into the polymer matrix. The structure and bulkiness of the monofunctional, bifunctional, and multifunctional organic ligands will control the surface area, the uniform pore size distribution on and within the polymer, and the geometry and configuration of the channels within the polymer. The number of oxo or nitro nucleophilic groups available for Pd coordination will determine the distribution of the Pd active sites on the solid support of the catalyst. These attributes conferred by the organic ligands to the Pd-Ln CPs will be important for achieving high activity and chemoselectivity, good stability, and reusability of the heterogeneous catalysts in Suzuki–Miyaura cross-coupling reactions. As illustrated in Table 1, 2,2′-bipyridyl-4,4′-dicarboxylic acid [123,124,125,126], 2,2′-bipyridyl-5,5′-dicarboxylic acid [127,128], and 1,3-bis(carboxymethyl)imidazolium ion [149] have been successfully associated with Pr, Gd, Tb, Nd, Ce, Eu, and Sm in providing superior Pd catalytic systems. In addition, new ligands containing nitrogen atoms along with carboxylic groups [129,130,136,137,138,139,140,141,142,143,144,145,146] expand the coordination possibilities for generating the targeted steric configuration of the coordination polymers. Complex multifunctional ligands like 3,3′,5,5′-azobenzenetetracarboxylic acid [146], 2,6-bis(2,4-dicarboxylphenyl)-4-(4-carboxylphenyl)pyridine [141,142], 4,4′,4″-(benzene-1,3,5-triyltris(azanediyl))tribenzoate [144], 1,3,5-benzene tricarboxylic acid [131], and 4,4′,4′′-nitrilotribenzoic acid [140] have been used to coordinate a broad range of lanthanides (e.g., Sm, Eu, Gd, Tb, Dy, Ho, Er, Tm, Yb, La, Lu, Nd, Ce, and Yb) in building new Ln coordination polymers with diverse configurations. It is worth mentioning that heterobimetallic coordination polymers incorporating the bifunctional organic ligand 1,1′-di(p-carboxybenzyl)-2,2′-diimidazole associated with Sm, Eu, Tb, and Dy and decorated with Pd catalytic sites produced high yields in the cross-coupling reaction of aryl bromides with arylboronic acids [145]. Also, Ln coordination polymers with polyfunctional tetracarboxylic ligand 3,3′,5,5′-azobenzenetetracarboxylic acid, associated with Ln^III^ ions (Ln = Sm, Eu, Gd, Tb, Dy, Ho, Er, Yb), resulted in quantitative yields in the related CO_2_ cycloaddition reaction with epichlorohydrin [146]. In some cases, mixed organic ligands [147] and bidentate or tetradentate metalloligands [127,136,147,148] have been used to build robust networks of multifunctional coordination polymers. By their stereochemical configuration and mode of coordination, the organic linkers [147] trigger stable structures and diverse topologies with uniformly distributed porosity and definite channels. Recently, research extended the array of organic ligands used for coordination polymers to various organophosphonates [89], organosulfur [147], and new nitrogen-containing compounds [147,148].

An important class of organic ligands employed in Pd-Ln coordination polymers consists of ancillary ligands coordinated to palladium with a predominant role in controlling and tuning the catalyst reactivity and selectivity. These ligands contribute to stabilizing the palladium active species and adjusting their electronic and steric properties. Frequently, phosphine ligands have been used in the Suzuki reaction because they increase the electron density at the palladium active site and therefore assist the oxidative addition step. Furthermore, the bulkiness of the substituents of the phosphine ligand favors the reductive elimination step of the catalytic cycle. Notwithstanding, due to the instability of the phosphine ligands, recently, *N*-heterocyclic carbenes (NHCs) have been preferred as efficient ligands in Pd complexes. *N*-Heterocyclic carbenes are more electron-rich and bulky than the phosphine ligands and, accordingly, the electronic and steric factors of the *N*-heterocyclic carbene ligands contribute substantially to stabilize the active Pd(0) sites [150]. Both types of ligands distinctly determine the activity of the palladium catalytic sites. It is noteworthy that the organic linkers endowed with oxo, nitro, or thio nucleophilic groups are able to increase the electron density at the Pd sites by charge back-donation. Moreover, they serve as real agents of charge transfer from highly electropositive lanthanides to palladium, enhancing further the stability of the palladium active species. On the other hand, the ancillary ligands bound to palladium, by their electronic and steric effects, directly adjust the activity and stability of the palladium catalytic species. In this context, we would like to mention that the substantial electron density and steric hindrance of NHC ligands are relevant for enhancing the oxidative addition and reductive elimination steps in palladium catalysis (Scheme 1).

## 4. New Applications of Pd-Ln CPs as Catalysts in Suzuki–Miyaura Cross-Coupling Reactions

**Figure 1:**
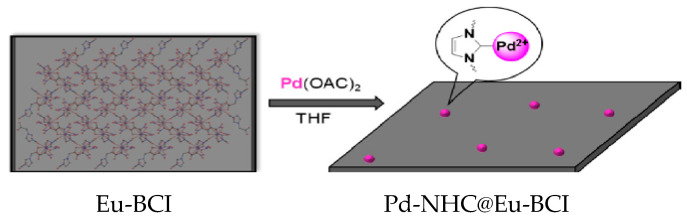
Two-dimensional configuration of Pd-NHC@Eu-BCI coordination polymers (adapted from Ref. [138]).

There are important applications of Pd-Ln coordination polymers as heterogeneous catalysts in Suzuki–Miyaura cross-coupling and related reactions. In a recent contribution, You et al. [138] reported on the synthesis of the NHC–Pd-functionalized coordination polymer (Pd-NHC@Eu-BCI) by a two-step procedure and its efficient application as heterogeneous catalyst for the cross-coupling reaction of aryl halides with aryl boronic acids. In their work, the new catalyst Pd-NHC@Eu-BCI was synthesized by incorporating N-heterocyclic carbene–palladium active sites into a 2D Eu-coordination polymer [Eu(BCI)(NO_3_)2H_2_O]n (Eu-BCI) by means of 1,3-bis(carboxymethyl)imidazolium (HBCI) as the bridging ligand. The active palladium sites were generated by the reaction with Pd(OAc)_2_, under appropriate conditions (molar ratio, solvent, temperature, and time). The two-dimensional configuration of the Pd-NHC@Eu-BCI coordination polymer stemmed from the bifunctionality of the organic ligand (HBCI) and coordination preference of europium (Figure 1).

The key role of bifunctional 1,3-bis(carboxymethyl)imidazolium (HBCI) in generating the NHC ligand and allowing a definite porosity of the CP matrix was essential for the catalytic activity. In addition, Eu ion ensured the good stability of the coordination polymer and a synergistic interaction with the Pd active sites. The catalyst was carefully characterized by different analytical techniques such as X-ray photoelectron spectroscopy (XPS), inductively coupled plasma atomic emission spectroscopy (ICP-AES), energy-dispersive X-ray spectroscopy (EDS), scanning electron microscopy (SEM), transmission electron microscopy (TEM), powder X-ray diffraction (PXRD), infrared spectroscopy (IR), and thermogravimetric analysis (TGA). It is important to mention that the XPS spectra revealed that all the palladium species within the catalyst were in the divalent oxidation state. The elemental composition of the catalyst was examined by energy-dispersive X-ray spectroscopy (EDS). The EDS results confirmed the presence of C, N, O, Eu, and Pd elements in the Pd-NHC@Eu-BCI coordination polymer. Interestingly, in the scanning electron microscopy (SEM) image, the authors observed that the catalyst was composed of irregular blocks with a size of about 2–20 µm. Furthermore, the transmission electron microscope (TEM) image indicated that there were no Pd nanoparticles incorporated into the polymer. Additionally, the elemental mapping images revealed the homogeneous dispersion of Pd, Eu, O, and N elements, confirming the rigorous synthesis procedure of the Pd-NHC@Eu-BCI coordination polymer. Significantly, a high catalytic activity of Pd-NHC@Eu-BCI (yields > 99%) was attained for the cross-coupling reaction of certain aryl halides with aryl boronic acids, under specific conditions. The best reaction conditions were when using K_2_CO_3_ as the base, ethanol as the solvent, and a catalyst loading of 25 mg Pd-NHC@Eu-BCI, at a temperature of 80 °C. A turnover number (TON) of 374 and a turnover frequency (TOF) of 62.3 h^−1^ were recorded. Interestingly, when Pd(OAc)_2_ was used under the optimal reaction conditions, the yield of biphenyl decreased to 87%. The experimental results also indicated quantitative yields for 4-Me as substituent on the aryl halide and boronic acid (99%), while with 4-COMe yields of 91–92% were attained, showing that the catalyst preferably promotes the reaction of substrates bearing electron-donating substituents. The catalyst from the reaction mixture was easily recovered by filtration and on recycling exhibited good catalytic activity for cross-coupling. Importantly, the catalyst maintained its original structure after five cycles. The above authors proposed a reaction mechanism for the cross-coupling reaction using the Pd-NHC@Eu-BCI catalyst that implies the oxidative addition of Pd(0) with aryl halide to form a Pd(II) complex which undergoes transmetalation with arylboronic acid after complexation with the base. In the subsequent step, by reductive elimination the biaryl product is formed and the Pd (0) complex is generated, prone to continue the catalytic cycle. The significant contribution of NHC as an electron-rich and bulky ligand in activating both the oxidative addition and reductive elimination steps in the Pd catalytic cycle was evidenced [150,151,152].

**Figure 2:**
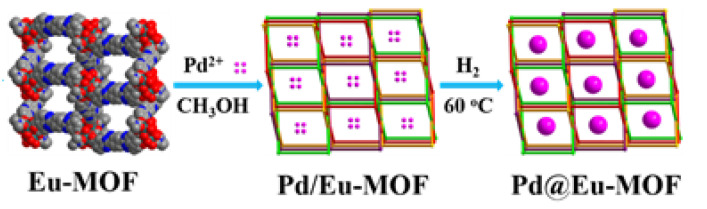
Three-dimensional configuration of Pd@Eu-MOF catalyst obtained by immobilization of Pd nanoparticles (Pd NPs) on Eu-MOF (adapted from Ref. [153]).

A novel catalyst based on Pd and Eu, Pd@Eu-MOF, was reported by Sun et al. [153] through the immobilization of Pd nanoparticles (Pd NPs) on the highly porous, hydrothermally stable Eu-MOF. The protocol applied in two steps consists of solution impregnation and H_2_ reduction. The sizes of nanoparticles ranged between 2 and 5 nm. (Figure 2).

In this procedure, the three-dimensional Eu-MOF {[Eu_2_(L)_3_(H_2_O)_2_(DMF)_2_]16H_2_O}*_n_*, a coordination polymer decorated with Pd nanoparticles, used 1,4-bis(5-carboxy-1*H*-benzimidazole-2-yl)benzene (H_2_L) as the organic ligand. The choice of the bridging ligand and europium in the construction of this type of catalyst was essential for creating adequate porosity, an appropriate surface, polymer matrix geometry, robustness, and hydrothermal stability in view of practical applications. The final composite material was fully characterized by appropriate analytical methods (SEM, EDS, ICP-OES, PXRD, and XPS). The H_2_ reduction approach allowed a new Pd@Eu-MOF catalyst to be obtained with Pd NPs sizes ranging between 2 and 5 nm. Detailed investigations by EDS, SEM, and mapping images of the nanocomposite structure showed that the elements of Eu, Pd, Cl, C, N, and O are properly distributed in the Pd@Eu-MOF nanocatalyst. Following PXRD examination of the Eu-MOF, the authors observed that this framework is chemically stable under aqueous sodium hydroxide and hydrochloric acid solutions (pH = 1–14) or in organic solvents frequently used in practical applications. The porosity of the coordination polymer was investigated by N_2_ sorption measurements at 77 K. The results indicated that Eu-MOF, Pd/Eu-MOF, and Pd@Eu-MOF display the typical type-I sorption isotherms, revealing that the microporous properties of Pd@Eu-MOF presented a slight decrease in the amount of adsorbed N_2_, as compared to Eu-MOF and Pd/Eu-MOF. In addition, the BET measurements of the surface area indicated a decrease from 1361 to 706 m^2^ g^−1^ after Pd nanoparticles had been loaded. The new Pd@Eu-MOF-based catalyst was successfully applied in two different catalytic reactions, i.e., Suzuki–Miyaura cross-coupling and addition of CO_2_ to epoxides. The dual functionality of Pd@Eu-MOF in two mechanistically distinct reactions was explained by taking into account the predominant role of the Lewis acid sites on the MOF in activating, at the same time, the rate-determining oxidative addition step of the palladium catalytic cycle in carbon–carbon cross-coupling reaction and the available epoxy ring for cycloaddition reaction. In both cases, the authors proved by PXRD that the stability of Pd@Eu-MOF catalyst was maintained after the reactions. It is noteworthy that in the present combination of Pd with Eu, cooperation between these two catalytic species became possible, enhancing the overall catalyst activity. In this connection, under the optimized reaction conditions, high yields have been attained (98–99%) in both catalytic processes, C(sp^2^)-C(sp^2^) cross-coupling and CO_2_ fixation. It is of interest that in Suzuki–Miyaura cross-coupling reactions of substrates with electron-donating substituents (4-Me) versus electron-withdrawing substituents (4-COMe) at the phenylboronic acid, higher yields were obtained for 4-Me (>99%) compared to 4-COMe (87%), suggesting that the catalyst is more prone to catalyze the reaction of phenylboronic acid-bearing electron-donating substituents. This outcome was rationalized considering essential the activating effect of the electron-donating group on the ligand exchange rate within the boron complex during the transmetalation step of the palladium catalytic cycle.

The heterogeneous catalyst was effectively recovered and efficiently reused in more than four catalytic cycles, indicating its proper stability and reproducibility. Detailed investigations on the recycled catalyst by PXRD, TEM, and XPS techniques evidenced that the Pd@Eu-MOF maintained its original structure and morphology. Also, during the catalyst recycling process, agglomeration of Pd nanoparticles was not detected, confirming the stability of the catalytic system.

Taking advantage of the metalloligand ability to complexation with Ln^3+^ ions, Jin and coworkers [127] reported a significant investigation on the coordination polymers containing various lanthanides (Sm, Eu, Gd, or Tb) and their fruitful application in Suzuki–Miyaura and Heck cross-coupling reactions. In this work, by an original and rather elaborate methodology, they succeeded in incorporating a [Pd(diimine)Cl_2_] scaffold into porous Ln-based heterometallic coordination polymers. The basic idea followed by the above authors was that introducing a soft nitrophilic metal cation may preferentially coordinate the diimine groups of the mixed donor ligand such as 2,2′-bipyridine-5,5′-dicarboxylic acid (H_2_bpydc). Thus, according to this protocol, in a first step, the Pd [Pd(H_2_bpydc)Cl_2_] metalloligand was produced and further complexed with different Ln^3+^ ions, to build, via a conventional hydrothermal procedure, the heterobinuclear coordination polymers {Ln_2_[Pd(bpydc)Cl_2_]_2_[Pd(Hbpydc)Cl_2_]_2_-(H_2_O)_4_}_n_ (Ln = Sm, Eu, Gd or Tb). The Ln-coordination polymers thus obtained are endowed with two types of functional metal centers, Ln^3+^ as a linker-bridging metal center and Pd^2+^ as a catalytic metal center. The cooperation between the two metal sites will enhance the catalytic activity of the heterobimetallic system. It is noteworthy that these porous materials using different lanthanides displayed isomorphism, with their structures crystallizing in the triclinic space group P1. When applied as heterogeneous catalysts in the Suzuki–Miyaura reaction of aryl derivatives with phenylboronic acid, under optimized molar ratios, in toluene, at 95 °C, for 4 h and employing K_2_CO_3_ as a base, they provided good yields (85 to 97%) in biaryl compounds. It is also noteworthy that these new heterogeneous palladium catalysts showed unusual versatility, catalyzing the Suzuki–Miyaura reaction with acceptable yields of coupling products and little wastage of the palladium.

**Figure 3:**
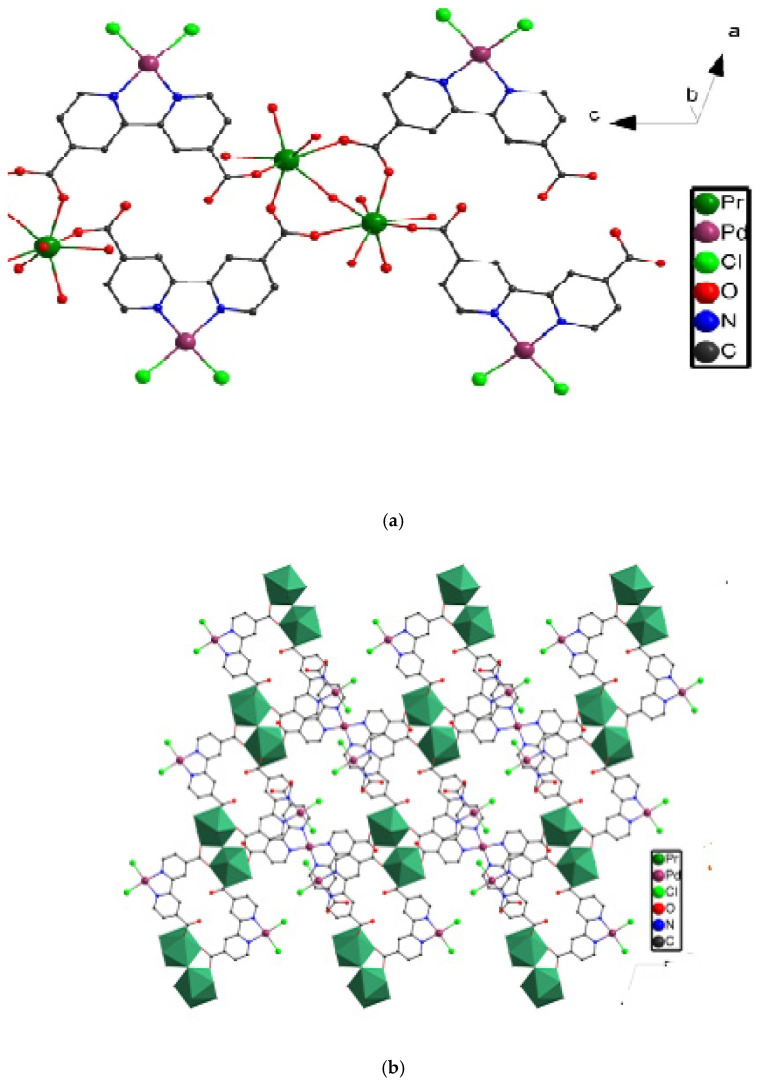
(**a**) One-dimensional layer of Pd-Pr coordination polymer (Copyright 2016, Elsevier). (**b**) Two-dimensional layer of Pd-Pr coordination polymer (Copyright 2016, Elsevier).

In a new contribution, Sun and coworkers [124] employed a set of lanthanides to conveniently prepare heterobimetallic coordination polymers, [Ln_2_Pd_3_(BPDC)_2_(HBPDC)_2_(*μ*_2_-O)Cl_4_(H_2_O)_6_·nH_2_O]*_m_* (Ln = Pr, n = 5, Ln = Gd, n = 4, and Ln = Tb, n = 4) endowed with high air and water stability. They designed a reticular synthesis approach, allowing coordination of the nitrophilic Pd(II) units and oxophilic Ln(III) ions through the heteroleptic ligand, 2,2′-bipyridine-4,4′-dicarboxylic acid (H_2_BPDC). The bifunctional organic linker was able to coordinate the lanthanides under common hydrothermal conditions, providing a coordination polymer with a distinct structure and configuration. These novel materials have been structurally characterized by single-crystal X-ray diffraction, FT-IR, powder X-ray diffraction, elemental analysis, and thermogravimetric (TG) techniques. The results indicated that the Pd-Ln coordination polymers obtained are isostructural and show similar structural characteristics. Single-crystal X-ray diffraction investigation showed that these heterobimetallic coordination polymers display similar 3D frameworks, connected via hydrogen bonding interactions. Three distinct types of building blocks were generated in the process: Pd(BPDC)Cl_2_, Pd(HBPDC)_2_, and an Ln dimer (Ln_2_O_15_). In the case of praseodymium, the building blocks with the structure Pd(HBPDC)_2_ further connected the Pr dimers into infinite 1D chains to construct a 2D layer in the *ac* plane (Figure 3a,b).

Figure 3a illustrates the coordination environment and geometrical arrangement of the Pr^3+^ and Pd^2+^ ions bridged by the organic ligand (2,2′-bipyridine-4,4′-dicarboxylate) in the one-dimensional layer of the Pd-Pr coordination polymer. The organic ligand bridging Pr with Pd, by virtue of its electronic structure and mobility of the aromatic moieties and carboxylate groups, has the ability to control the charge transfer from the electropositive Pr ion toward Pd ion. This effect of the lanthanide, facilitated by the organic ligand as the charge transfer agent, will enhance the reduction process of Pd(II) to Pd(0). As a result, the lanthanide will accelerate by this kinetic effect the reductive elimination step of the palladium catalytic cycle and will increase the concentration of Pd(0), able to resume a new oxidative addition process (Scheme 1). This example clearly demonstrates the beneficial cooperative effect of lanthanide on the palladium cross-coupling reactions, governed by the electropositive propensity of lanthanide and the electronic structure and mobility of the organic ligand as the charge transfer vector [124]. Furthermore, the 2D layers (Figure 3b) are assembled into a 3D framework via hydrogen bonding interactions, building a three-dimensional polymer with adequate porosity which is prone to catalytic reactions. This set of coordination polymers was effectively employed as heterogeneous catalysts in Suzuki–Miyaura, Heck, and Sonogashira cross-coupling reactions. Specifically, when applied in Suzuki–Miyaura cross-couplings of aryl halides with arylboronic acids, these isostructural coordination polymers exhibited good activity (yield > 90%), working under green, eco-friendly reaction conditions. Notably, the best results were obtained for the Pd-Pr catalyst in DMF/H_2_O as solvent, using K_2_CO_3_ as base, at 70 °C optimal temperature (Table 2, Ln = Pr, Gd, Tb).

The beneficial cooperation between lanthanides and palladium was also assumed to be present in this catalytic process. The sustainability, utility, and cost-effectiveness of this approach have been demonstrated by performing recycling experiments, proceeding practically without loss of catalyst activity after four runs in Suzuki–Miyaura cross-couplings.

In a detailed study on the Suzuki–Miyaura cross-coupling reaction, Ding and coworkers [128] designed an attractive and productive one-pot procedure to prepare an array of heterodinuclear Pd-Ln complexes, with the formulas Pd-bpydc-Nd, Pd-bpydc-Ce, and Pd-bpydc-La (bpydc = 2,2′-bipyridine-5,5′-dicarboxylate). These Pd-Ln complexes were effectively applied as catalysts in the cross-coupling of *p*-bromoanisole with phenylboronic acid under eco-friendly reaction conditions. As shown in Table 3, the Pd-bpydc-Nd catalyst provided the most interesting results (Table 3).

**Scheme 2:**
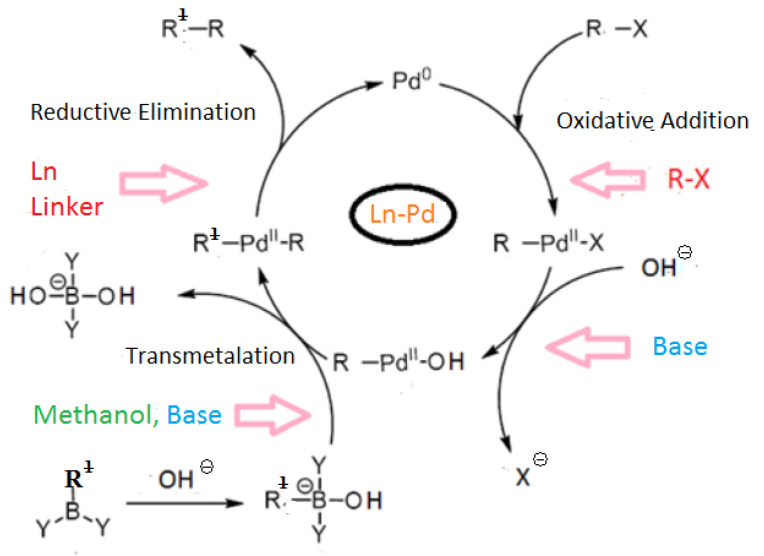
Influence of lanthanides (Ln = La, Ce, Nd), substrate, base, and solvent on Pd catalytic cycle in Suzuki–Miyaura cross-coupling reaction (adapted from Ref. [128]).

The above authors on purpose selected Nd, Ce, and La as the rare-earth elements to identify the effect of the lanthanide’s nature on the catalyst’s performance, and indeed they obtained satisfying results. It is important to note that the difference in activity and selectivity generated by the isostructural Nd, Ce, and La complexes in the catalytic systems was assigned to the nature of the lanthanide, with Nd giving the best outcome, and not to the crystal structure which is similar for this set of lanthanides. A significant dependence on the catalyst loadings was identified. The majority of the experimental tests were carried out in the temperature range of 30–60 °C, with catalyst/substrate molar ratios corresponding to different catalyst loadings (varying from 0.5 mmol% to 0.2 mmol%) and choosing the sodium tert-butoxide–methanol pair as the base and solvent. This pair of components ensured the good solubility of the reaction partners and intermediates in the reaction medium. It is noteworthy that with low catalyst loadings, substantial yields (93–95%) and conversions (73–99%) have been achieved when working under optimal temperatures and times. The yields and conversions followed the order Pd-bpydc-Nd > Pd-bpydc-Ce > Pd-bpydc-La. The role of the lanthanide in directing the catalyst performance was relevant. Nd was the best from the set of lanthanides employed for building the catalyst. Importantly, an increase in the conversion and yield with increasing temperature was found to be a general trend in this cross-coupling reaction for all the catalysts employed. Notwithstanding, the most advantageous yields were attained at a temperature of 60 °C, which is lower than the frequently used temperatures for the cross-coupling reactions in common organic solvents. Based on the above results, Ding et al. [128] considered a mechanistic model which complies well with the general reaction mechanism accepted for Suzuki–Miyaura reactions [RX + R^1^B(OH)_2_]. Essentially, the Pd catalytic cycle of this reaction involves oxidative addition [Pd(0) to RPd(II)X], exchange reaction (metathesis) between RPd(II)X and base MOH to RPd(II)OH, the transmetalation of RPd(II)OH with boron complex [R^1^BY_2_OH]^--^ to R^1^Pd(II)R and the reductive elimination of R^1^Pd(II)R to Pd(0) and R^1^—R (Scheme 2).

The role of the base in activating the arylboronic acid in the transmetalation step of the palladium catalytic cycle was in agreement with the data recorded so far in this field [154,155,156,157]. Additionally, the selection of a proper base–solvent system will improve the base and aryl halide solubility with a favorable outcome for the cross-coupling reaction [158,159,160]. Importantly, the above authors showed that the influence of the lanthanides on the reductive elimination step of the palladium catalytic cycle is distinct from that exerted by the base, solvent, and substrate. Essentially, the base and solvent determine mainly the transmetalation step while the substrate influences by its electronic effect the oxidative addition step of the palladium catalytic cycle. In addition, lanthanides have a specific influence on the reductive elimination step. In the present case, Nd^3+^, endowed with a small ionic radius and ready availability of f electrons (Table 4), appears to have a predominant influence on the Pd site through charge transfer, mediated by the flexible organic linker, resulting in an enhancement of the reductive elimination step of the Pd catalytic cycle. This process occurs via a significant cooperation between the lanthanide and the aromatic linker in transferring charge density to the nitrogen–Pd bond.

An interesting array of chelating-amino-functionalized lanthanide metal–organic frameworks, Y-DDQ, Dy-DDQ, and Eu-DDQ, has been published by Zhu et al. [143]. They used a flexible dicarboxylate ligand derived from quinoxaline (H_2_DDQ  =  *N*, *N*′-dibenzoic acid-2,3-diaminoquinoxaline). These authors demonstrated that the new 3D frameworks are built up through the *N*, *N*′-dibenzoic acid-2,3-diaminoquinoxaline (H_2_DDQ) linker that succeeds in connecting a special type of zigzag ladder configuration. In this way, they generated a net of sra topology within their structures. They showed that the Ln(III) ionic center is six-coordinated, creating an open metal site, whereas the free chelating amino groups behave as free functional organic sites. It is noteworthy that by N_2_ and Ar adsorption studies, the authors proved that the Ln-DDQ exhibits stable microporous frameworks, displaying high surface areas even after the removal of the solvent. Following an appropriate post-synthetic modification protocol, the Pd nanoparticles were immobilized onto the MOFs by using the graft interactions between free chelating amino groups and the metal ions. The well-dispersed Pd@Ln-DDQs nanomaterials prepared in this way have been suitable catalysts for Suzuki–Miyaura cross-coupling reactions of aryl halides with arylboronic acids. Using a rather simple methodology, Sun and coworkers [130] published the synthesis of a new, highly active, magnetically recoverable, and recyclable heterogeneous catalyst applied successfully in Suzuki–Miyaura cross-coupling reactions. Essentially, the catalyst Fe_3_O_4_@La-MOF-Schiff-base-Pd, based on La-MOF support, contains Fe_3_O_4_ nanoparticles to convey magnetic properties to the compound with a Schiff base for Pd derivatization and immobilized Pd nanoparticles as catalytically active sites. In their protocol, they used efficiently Pd nanoparticles (NPs) for the post-synthetic modification (PSM) of La-MOF. In a first step, the La-MOF [La(abdc)(Habdc)·9H_2_O]_∞_ was prepared using 2-amino-benzene-1,4-dicarboxylate (abdc) as the organic ligand and then conveniently immobilized Fe_3_O_4_ NPs on this framework. The authors took advantage of the 3D structure of the La-MOF support ensuring an adequate channel geometry of the matrix cavity, uniform porosity, and proper stability, suitable for catalytic applications. The appropriate porosity, cavity, and channel configuration of La-MOF obtained from 2-amino-benzene-1,4-dicarboxylate and the better stability of the heterogeneous catalyst to solvents and chemical reagents were the main attributes for using La-MOF as support for PSM. As an advantage, the method for synthesizing the La-MOF was rather straightforward so that it can be valorized on a large-scale. In the methodology employed, the Fe_3_O_4_@La-MOF coordination polymer was prepared containing in advance Fe_3_O_4_ nanoparticles and then post-synthetically modified by reaction with pyridine-2-carboxaldehyde to finally obtain the imine derivative Fe_3_O_4_@La-MOF-Schiff-base. The Schiff-base-containing composite was subsequently combined with PdCl_2_ to produce the Pd-functionalized framework Fe_3_O_4_@La-MOF-Schiff-base-Pd. By carefully examining the product through PXRD, it was proved that Fe_3_O_4_ nanoparticles were properly immobilized on the La-MOF template. The crystalline state of the backbone was shown to preserve well during the post-modification step. The catalytic activity of the Fe_3_O_4_@La-MOF-Schiff-base-Pd was thoroughly investigated in the Suzuki–Miyaura cross-coupling of aryl halides with aromatic boronic acids. Relevant results are presented in Table 5.

Experiments with the above heterogeneous catalyst Fe_3_O_4_@La-MOF-Schiff-base-Pd revealed that it displays significant activity and chemoselectivity, providing appreciable TONs and TOFs in Suzuki–Miyaura cross-coupling, and, importantly, these results are obtained at low catalyst loadings. Data evidenced that the best combination providing good yields was using K_2_CO_3_ as the base and ethanol as the solvent. On raising the reaction temperature, the yield sharply increased. At a temperature of 80 °C and a reaction time of 0.5 h, the yield was as high as 99%. An efficient synergistic interaction between Pd and La, promoted by both the Shiff base and the 2-amino-benzene-1,4-dicarboxylate ligand incorporated into the MOF template, was evidenced. The preferential cooperation of palladium with transition metals is well known in palladium catalysis [162]. Furthermore, in this approach, the Fe_3_O_4_@La-MOF-Schiff-base-Pd was quite stable and could be conveniently recovered magnetically and reused in cross-coupling reactions. Notably, the catalyst was recycled 12 times and retained a consistent level of its catalytic activity after recycling.

Overall, as illustrated in Table 2, Table 3, and Table 5, in connection with Equations in them, respectively, the distinguishing features of the Pd–Ln catalytic systems relative to the traditional Pd-based catalysts consist in high activity and chemoselectivity, catalyst stability and recyclability, and specific reaction conditions (solvent, base, temperature, and catalyst loading). Whereas the activity and chemoselectivity, catalyst stability, and recyclability are high and advantageous in all cases, the difference in the appropriate solvent is evident, with the [Ln_2_Pd_3_(BPDC)_2_(HBPDC)_2_(*μ*_2_-O)Cl_4_(H_2_O)_6_·nH_2_O]*_m_* (Ln = Pr, Gd, Tb) catalyst favoring better an aqueous system (DMF-H_2_O) (Table 2), the Pd-bpydc-Nd, Pd-bpydc-Ce, and Pd-bpydc-La needing methanol as solvent (Table 3), and Fe_3_O_4_@La-MOF-Schiff-base-Pd using ethanol as solvent (Table 5). The low catalyst loadings (0.066 mmol% Pd) and high recyclability (12 times) of the last catalytic system in this series are remarkable, as compared to the conventional catalytic systems applied in Suzuki–Miyaura cross-coupling reactions [17,18].

## 5. Conclusions

Developments in Pd-Ln coordination polymers illustrate that this class of heterogeneous catalysts is highly active and chemoselective in Suzuki–Miyaura C(sp^2^)-C(sp^2^) cross-coupling reactions. The selection of a wide range of lanthanides, e.g., Eu, Sm, Eu, Gd, Pr, Nd, Ce, La, or Tb, and various organic linkers such as 2,2′-bipyridyl-4,4′-dicarboxylic acid, 2,2′-bipyridyl-5,5′-dicarboxylic acid, and 1,3-bis(carboxymethyl)imidazolium ion, allowed the construction of Pd-Ln coordination polymers endowed with advantageous physical–chemical characteristics (i.e., a large surface area, a uniform distribution of pores, suitable geometry and configuration of channels, acceptable robustness, and proper adjustment of active sites) to be applied as heterogeneous catalysts in C(sp^2^)-C(sp^2^) cross-coupling reactions. Due to their multiple coordination numbers, lanthanides permitted the construction of two- and three-dimensional architectures with convenient cavities to host the active palladium sites. Their substantial oxophilic character and hard Lewis acid propensity favored the formation of stable structures of coordination polymers and viable matrices via tightly bridging lanthanides through the coordinating groups of the organic linkers. Moreover, due to their electropositive propensity, lanthanide ions influence the coordinative palladium bonds, enhancing the back-donation charge distribution and generating a rather stable Pd complex. The effect of lanthanide ions and ancillary ligands associated with palladium on the oxidative addition and reductive elimination steps of the palladium catalytic cycle has been clearly outlined. Detailed studies on the Pd-Ln coordination polymers as catalysts in cross-coupling reactions convincingly evidenced that lanthanides cooperate in a synergistic way with palladium, thus improving the activity and chemoselectivity of the catalytic sites. The essential role of the organic linkers bridging lanthanides within the polymer matrix in creating robust and stable configurations of coordination polymers has been fully illustrated. Moreover, the considerable potential of Pd-Ln coordination polymers for future applications as sustainable heterogeneous catalysts in carbon–carbon cross-couplings and related reactions has been demonstrated.

## Figures and Tables

**Table 1:** Organic ligands for catalytic Pd-Ln coordination polymers (Refs. [123,124,125,126,127,128,129,130,131,132,133,134,135,136,137,138,139,140,141,142,143,144,145,146,147,148,149]).

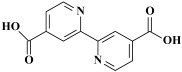				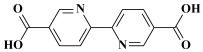				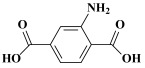			
(Refs. [123,124,125,126])				(Refs. [127,128])				(Refs. [129,130])			
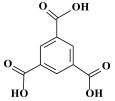			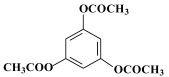			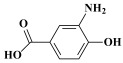			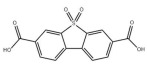		
(Ref. [131])			(Ref. [132])			(Ref. [133])			(Ref. [134])		
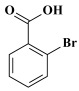			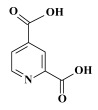			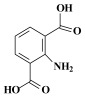			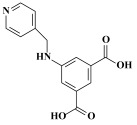		
(Ref. [135])			(Ref. [136])			(Ref. [129])			(Ref. [137])		
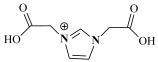						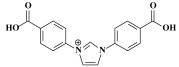					
(Ref. [138])						(Ref. [139])					
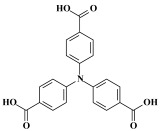				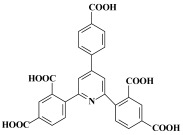				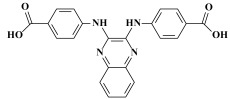			
(Ref. [140])				(Refs. [141,142])				(Ref. [143])			
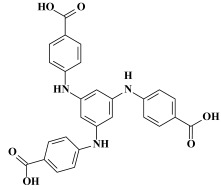				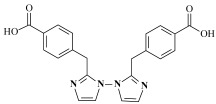				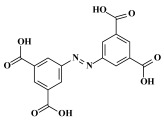			
(Ref. [144])				(Ref. [145])				(Ref. [146])			
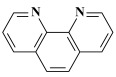				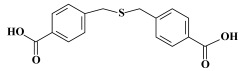				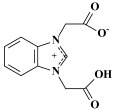			
(Refs. [147,148])				(Ref. [147])				(Ref. [149])			

**Table 2:** Suzuki–Miyaura cross-coupling reaction of aryl halides and arylboronic acids in presence of Pd-Ln (Ln = Pr, Gd, Tb) catalysts ^a^.

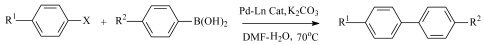					
**Entry**	**R^1^**	**R^2^**	**X**	**Catalyst**	**Yield (%) ^b^**
1	CH_3_CO	H	Br	Pd–Pr	91
2	CH_3_CO	H	Br	Pd–Gd	80
3	CH_3_CO	H	Br	Pd–Tb	82
4	NO_2_	H	Br	Pd–Pr	89
5	H	H	Br	Pd–Pr	86
6	H	H	I	Pd–Pr	80
7	CH_3_	H	I	Pd–Pr	90
8	CH_3_CO	H	I	Pd–Pr	93
9	H	CH_3_	Br	Pd–Pr	91
10	CH_3_CO	CH_3_	Br	Pd–Pr	92
11	NO_2_	CH_3_	Br	Pd–Pr	94
12	H	CH_3_CO	Br	Pd–Pr	89
13	CH_3_CO	CH_3_CO	Br	Pd–Pr	70
14	CH_3_CO	H	Cl	Pd–Pr	22

^a^ Reaction conditions: aryl halide (1 mmol), arylboronic acid (1.5 mmol), K_2_CO_3_ (2.0 mmol), DMF-H_2_O (1:1, 6 mL), catalyst (0.4 mol%), 70 °C, 3 h. ^b^ Yields were determined by GC and GC–MS analysis (adapted from Ref. [124]).

**Table 3:** Suzuki–Miyaura cross-coupling of *p*-bromoanisole with phenylboronic acid in methanol induced by Pd-bpydc-Ln catalysts (Ln = Nd, Ce, La).

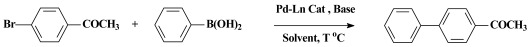				
**Entry**	**Catalyst**	**Temperature, °C**	**Time, h**	**Yield, % ^a,b,c^**
1	Pd-bpydc-Nd	30	8	95.0 ^a^
2	Pd-bpydc-Nd	30	8	93.4 ^b^
3	Pd-bpydc-Nd	60	4	95.0 ^a^
4	Pd-bpydc-Nd	60	4	94.9 ^b^
5	Pd-bpydc-Ce	30	8	25.0 ^a^
6	Pd-bpydc-Ce	30	8	25.9 ^b^
7	Pd-bpydc-Ce	60	4	91.9 ^a^
8	Pd-bpydc-Ce	60	4	88.9 ^b^
9	Pd-bpydc-La	30	8	35.0 ^a^
10	Pd-bpydc-La	30	8	15.0 ^b^
11	Pd-bpydc-La	60	4	55.9 ^a^
12	Pd-bpydc-La	60	4	14.9 ^b^

^a^ Reaction conditions: *p*-bromoanisole (0.5 mmol), phenylboronic acid (0.6 mmol), sodium tert-butoxide (0.5 mmol), methanol (1 mL), catalyst (0.5 mol %). ^b^ Catalyst (0.2 mol %). ^c^ Reaction yield (%) for total conversion determined from the internal standard yield, at the specified reaction time, based on GC, using hexadecane as internal standard (adapted from Ref. [128]).

**Table 4:** Ln3+ radius (pm) and electron configuration of La, Ce, and Nd a.

Lanthanide	La	Ce	Nd
Ln^3+^ radius (pm) ^a^	103	102	98.3
Ln^3+^ electron configuration ^b^	4f^0^	4f^1^	4f^3^

^a^ Refs. [111,161]; ^b^ Refs. [112,161].

**Table 5:** Suzuki–Miyaura cross-coupling reactions of various aryl halides with arylboronic acids using Fe_3_O_4_@La-MOF-Schiff-base-Pd as catalyst ^a,b,c^.

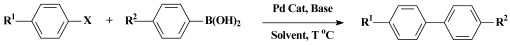				
**Entry**	**R^1^**	**X**	**R^2^**	**Yield, % ^a,b,c^**
1	H	I	H	>99
2	CH_3_	I	H	99
3	CH_3_CO	I	H	>99
4	H	Br	H	99
5	CH_3_	Br	H	95
6	CH_3_CO	Br	H	>99
7 ^b^	H	Cl	H	5
8 ^b^	CH_3_	Cl	H	14
9 ^b^	CH_3_CO	Cl	H	19
10 ^c^	H	Br	H	88
11	H	Br	CH_3_	99
12	H	Br	CH_3_CO	52

^a^ Reaction conditions: aryl halide (1.0 mmol), arylboronic acid (1.2 mmol), K_2_CO_3_ (2.0 mmol), ethanol (6 mL), catalyst (8 mg, 0.066 mmol% Pd), temperature (80 °C), time (0.5 h). ^b^ Reaction conditions: catalyst (10 mg), temperature (80 °C), time (10 h). ^c^ Reaction conditions: bromobenzene (2.0 mmol), phenylboronic (2.4 mmol), K_2_CO_3_ (4.0 mmol), ethanol (12 mL), catalyst (1 mg, 8.23 × 10^−3^ mmol% Pd), temperature (80 °C), time (0.5 h) (adapted from Ref. [130]).

## Data Availability

No new data were created or analyzed in this study.
